# A Joint 2D-3D Complementary Network for Stereo Matching

**DOI:** 10.3390/s21041430

**Published:** 2021-02-18

**Authors:** Xiaogang Jia, Wei Chen, Zhengfa Liang, Xin Luo, Mingfei Wu, Chen Li, Yulin He, Yusong Tan, Libo Huang

**Affiliations:** College of Computer, National University of Defense Technology, Changsha 410073, China; jiaxiaogang@nudt.edu.cn (X.J.); liangzhengfa10@nudt.edu.cn (Z.L.); luoxin13@nudt.edu.cn (X.L.); wumingfei10@nudt.edu.cn (M.W.); lichen14@nudt.edu.cn (C.L.); hylu@nudt.edu.cn (Y.H.); ystan@nudt.edu.cn (Y.T.); libohuang@nudt.edu.cn (L.H.)

**Keywords:** stereo matching, depth estimation, computer vision

## Abstract

Stereo matching is an important research field of computer vision. Due to the dimension of cost aggregation, current neural network-based stereo methods are difficult to trade-off speed and accuracy. To this end, we integrate fast 2D stereo methods with accurate 3D networks to improve performance and reduce running time. We leverage a 2D encoder-decoder network to generate a rough disparity map and construct a disparity range to guide the 3D aggregation network, which can significantly improve the accuracy and reduce the computational cost. We use a stacked hourglass structure to refine the disparity from coarse to fine. We evaluated our method on three public datasets. According to the KITTI official website results, Our network can generate an accurate result in 80 ms on a modern GPU. Compared to other 2D stereo networks (AANet, DeepPruner, FADNet, etc.), our network has a big improvement in accuracy. Meanwhile, it is significantly faster than other 3D stereo networks (5× than PSMNet, 7.5× than CSN and 22.5× than GANet, etc.), demonstrating the effectiveness of our method.

## 1. Introduction

Stereo matching is the progress of getting the depth information from stereo image pairs in the same scene, which is essential for Autonomous Driving [1], 3D Reconstruction and Mapping [2], Human-Computer Interaction [3], Marine Science and Systems [4], Planetary Exploration [5], Unmanned Aerial Vehicles (UAV) [6] or Person Re-identification [7,8]. Compared with expensive lidar equipment, stereo matching is convenient and high-efficient. Traditional methods [9,10,11,12,13,14,15,16] are mainly based on the brightness, color, and gradient of the image pairs, such as Census transform [12,13], Rank transform [15] and Birchfield and Tomasi [16], etc. Although they can get a rough disparity result at a fast speed, it is difficult for them to match the weak texture and occlusion areas. As a result, current stereo methods generally use convolutional neural networks (CNN) to improve performance.

MC-CNN [17] first replaces the traditional methods with a neural network in matching cost computation step and achieves a big improvement in accuracy. Thus most recent stereo methods use self-designed networks to compute matching costs (also called cost volumes).

**Figure 1:**
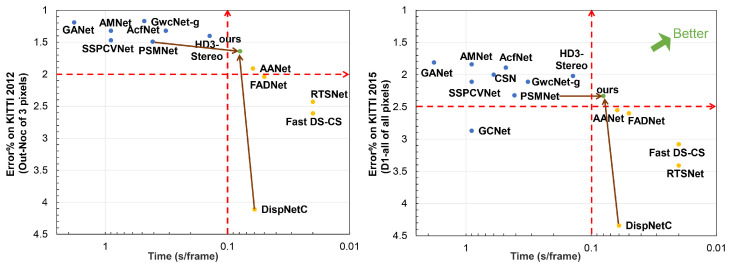
The visualization of speed and accuracy for recent stereo methods. We select the main evaluation metric for comparison from the KITTI official website. The smaller the *x* coordinate value, the faster the speed. The smaller the *y* coordinate value, the higher the accuracy. Blue dots represent accurate 3D networks, while yellow dots represent fast 2D networks. The green dot is our proposed method, a high-precision network with fast speed.

In terms of different matching cost computation methods, current neural network-based stereo methods can be mainly divided into the following: 2D networks [18,19,20,21,22,23] with cost volumes generated by traditional methods or the correlation layer. 3D networks [24,25,26,27] with cost volumes generated by concatenation. According to published papers on KITTI official website, these two architectures have obvious differences in speed and accuracy, as shown in Figure 1.

It is obvious from the figure that 2D networks have high speed but low accuracy, with running time is less than 100 ms and D1-all (percentage of stereo disparity outliers in all pixels) exceeds 2.50% for KITTI 2015. On the other hand, 3D networks have great advantages in accuracy, but the speed is low. Their running times exceed 100 ms, but D1-all are less than 2.50%. This is because the correlation layer loses much feature information when generating the cost volume. Both left and right features are converted into a value to represent the similarity between pixels. Meanwhile, the memory and running time grow cubically as the network dimension increases. Many methods also propose some special modules, leading to high accuracy and expensive computational cost. Therefore, it is difficult for current stereo networks to make a balance between speed and accuracy.

This paper analyzes the advantages and disadvantages of 2D and 3D stereo networks. Since 2D networks are fast with low accuracy, we first construct a 2D encoder-decoder to generate a rough disparity map quickly. Then we leverage an elaborate 3D stacked hourglass network to refine the disparity map from coarse to fine. In particular, the rough disparity map from the 2D network is expanded to a disparity range to guide the 3D network and reduce the computational cost. By the above steps, we can leverage the 2D and 3D networks to trade-off speed with accuracy, as shown in Figure 1.

The main contributions are listed as follows:

- We propose a complementary stereo method by integrating the advantages of 2D and 3D stereo networks. Our network can improve the accuracy of the 2D network and reduce the running time for the 3D network.
- We propose a fast and accurate stereo network, which can quickly generate an accurate disparity result in 80 ms.

## 2. Related Work

Stereo matching algorithms generally consist of four steps [28]: matching cost computation, cost aggregation, disparity computation, and refinement. In terms of matching cost computation methods, stereo networks can be divided into 3D cost volumes with 2D networks for cost aggregation and 4D cost volumes with 3D networks for cost aggregation.

Mayer et al. [22] introduce the correlation layer in the matching cost computation step, which is widely used in many 2D stereo networks. Specifically, after extracting features from stereo image pairs by a weight-sharing siamese network, the correlation layer uses a dot product style operation to decimate the feature dimension and summary the result into a similarity value. The final cost volume is a DSI (Disparity Space Image) 3D vector (H × W × D). Thus 2D convolutional networks is required to regularize and regress the depth or disparity. The correlation layer is defined as:

(1) $$C\left(d,x,y\right)=\frac{1}{N}\langle f_{l}\left(x,y\right),f_{r}\left(x,y-d\right)\rangle$$

where f(x,y) denotes the feature vector captured by neural network at the pixel (x,y). 〈〉 is the dot product for the left and right feature vectors. *N* is the number of channels.

Recent AANet [18] uses the correlation layer to generate multi-scale 3D cost volumes and uses the deformable convolution to construct AAModule for cost aggregation, which can achieve fast and accurate stereo results.

Although 2D networks can generate a rough disparity map with a fast running time and low memory cost, they still have much room for accuracy improvement. The correlation layer losses a lot of details of feature channels when generating the cost volume. To avoid this problem, current stereo methods mainly use 4D cost volumes and 3D convolution networks to reduce feature loss and improve accuracy.

Kendall et al. [24] replace the correlation layer by the concatenation operation to construct the 4D cost volume. Concretely, after extracting image features by a siamese network, they concatenate each unary feature of reference pixels with their corresponding unary from candidate pixels across each disparity level and packing these into a 4D volume (H × W × D × C). Then 3D convolutions are required for cost aggregation. The concatenation operation is stated in Equation (2):

(2) $$C\left(d,x,y\right)=Concat\left\{f_{l}\left(x,y\right),f_{r}\left(x,y-d\right)\right\}$$

This method is widely used in many stereo networks. For example, Chang et al. [25] propose PSMNet, which uses the SPP [29,30] module to extract image pair features and concatenates the features to construct a 4D cost volume. Then they design a stacked hourglass 3D network for cost aggregation. However, since 3D convolutions need expensive computational and memory cost, PSMNet takes 0.41 s to process a single stereo pair, which is an obstacle to deploy in real-time applications.

Researchers have proposed some methods to reduce the dimension of 4D cost volume (H × W × D × C) while maintaining accuracy to address this problem. For the C channel, GwcNet [31] combines the correlation layer and concatenation operation to generate a refined high-quality cost volume. NLCA-Net [32] replaces the concatenation operation by calculating the variance of extracted features, which can reduce the C channel by half. For the D channel, recent CSN [26] reduces this dimension by generating a disparity candidate range and gradually refining the disparity map in a coarse-to-fine manner. These methods can reduce the memory and computational cost to a certain extent. However, the fastest GwcNet also requires 0.32 s, only a small improvement for time (PSMNet 0.41 s → GwcNet 0.32 s).

In summary, 2D and 3D stereo networks have their own advantages and disadvantages in speed or accuracy. Meanwhile, there are some efficient 2D-3D and 3D-2D-3D algorithms used in different problems. For instance, Melo et al. [33] propose a proposition of an innovative method for estimating point cloud movements or deformation based on 2D/3D. Similar to this method, we make full use of the advantages of these two methods to construct a fast and accurate stereo matching network. We use the 2D network to generate a disparity range to guide and reduce the dimension of the 3D network. Meanwhile, we leverage the 3D network to improve the accuracy of the 2D network. These two networks are complementary in our paper. Thus we can achieve a balance between speed and accuracy, as shown in Figure 1.

## 3. Method

**Figure 2:**
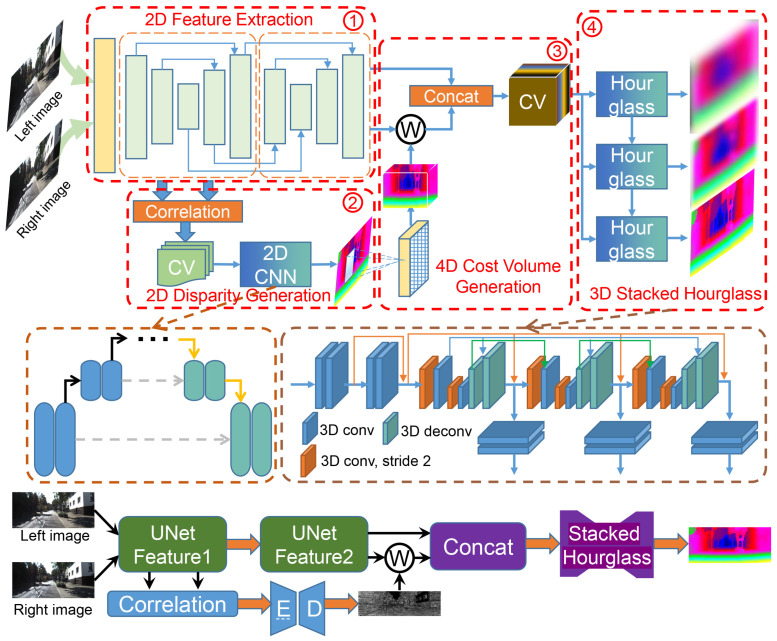
Overview of our proposed JDCNet. The stereo input images are fed to two cascaded Unet networks to capture features (step 1). Then we use the correlation layer with the first output features to generate the 3D cost volume and fed it into a 2D encoder-decoder for disparity regularization and regression (step 2). This rough disparity map can generate a disparity range to guide and reduce the 4D cost volume dimension (step 3). Finally, we leverage a 3D stacked hourglass network to generate the dense disparity result (step 4).

We propose JDCNet, an end-to-end stereo matching network from coarse to fine without any post-processing. It mainly includes the following modules: 2D feature extraction module, 2D disparity generation module, 4D cost volume generation module and 3D stacked hourglass module, as shown in Figure 2.

### 3.1. Network Architecture

The parameters of the proposed JDCNet are summarized in Table 1. The stereo images are fed into two cascaded Unet to extract features. Afterward, the first features are followed by the correlation layer to generate the 3D cost volume and 2D encoder-decoder for cost aggregation. The rough disparity map is converted into a disparity range to guide and reduce the subsequent 3D stacked hourglass network’s computational cost. Finally, our network can generate a dense disparity map from coarse to fine. Later sections will describe the details of all the parts of the proposed JDCNet.

### 3.2. 2D Feature Extraction Module

**Figure 3:**
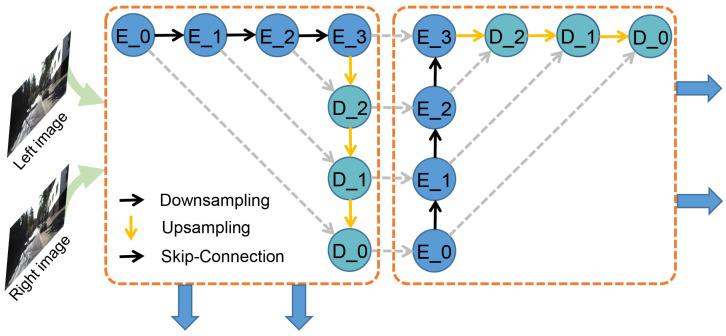
The details of feature extraction. Encoder (E_0, …, E_3), decoder (D_0, D_1, D_2) and Unet are connected by skip-connections. The first ouput feature is used for the correlation layer and 2D network, while the second is used for concatenation and 3D hourglass network.

We begin by downsampling the stereo pairs to 1/3 resolution with 5 × 5 kernel and 32 channel output. Then we construct two cascaded Unet [34] (an encoder-decoder with skip-connections and learnable parameters) to extract image pair features for both 2D and 3D networks. Each feature extraction network consists of 4 down-sampling layers for the encoder and four up-sampling layers for the decoder–all with 3 × 3 kernels, batch normalization and Relu layer. The channels only increase 16 at each scale to reduce computational and memory cost. The maximum resolution is 1/3 and the minimum is 1/48. The first decoder’s output will be used as the next encoder’s input, as shown in Figure 3, which can maximize the final receptive field. The dimensions of the final two features for 2D and 3D networks are 1/3H × 1/3W × 32. The first features are used for the correlation layer and generating a rough disparity map, while the second is used to generate the final accurate and dense disparity result.

### 3.3. 2D Disparity Generation Module

Since 2D networks are fast and memory friendly, we first use a 2D network to generate a rough disparity map quickly. After capturing the stereo pairs’ features, we use the correlation layer to construct a 3D cost volume. According to experience, the maximum disparity range is set to 192. Thus for 1/3 resolution, we can get a feature vector with dimension 1/3H × 1/3W × 64.

Similarly, we use a simple and efficient encoder-decoder structure as the cost aggregation network, which consists of five down-sampling, up-sampling layers and skip-connections. The channels keep 64 at each scale to reduce computational cost. We use trilinear interpolation to upsample the final cost volume to the original resolution (H × W × 192) and use it for disparity regression. Finally, we can get a rough disparity map with a running time of about 20 ms.

### 3.4. 4D Cost Volume Generation Module

We leverage the above disparity map to guide the 4D cost volume generation. We reduce the D channel of the cost volume by narrowing the disparity range, which can also reduce the parameters and running time. As mentioned by CSN, the disparity range of any pixel is determined by its surrounding pixels. We calculate the maximum and minimum values of the surroundings and compare them with the threshold σ=6 to get the reference pixel’s disparity range, as summarized in Algorithm 1.
**Algorithm 1** Calculating the disparity range.**Input:**       Rough disparity map generated by 2D network: $D_{i}$;**Output:**       Disparity range: $D_{r}$;  1:  Initialize:       Downsample the input to 1/4 resolution by bilinear interpolation: $D_{1/4}=BI\left(D_{i},1/4\right)$;   2:  $D_{max}=Max\_Pool\left(D_{1/4},kernel=3\right)$
  3:  $D_{min}=\left|Max\_Pool\left(-D_{1/4},kernel=3\right)\right|$
  4:  Use the threshold σ to adjust the disparity range:       $D_{Nmax}=\frac{D_{max}+D_{min}+\sigma }{2}$
       $D_{Nmin}=\frac{D_{max}+D_{min}-\sigma }{2}$
  5:  $D_{inter}=D_{Nmax}-D_{Nmin}/\left(\sigma -1\right)$
  6:  $D_{r}=D_{inter}\times arange\left(0,\sigma \right)+D_{Nmin}$
  7:  Upsample the output to original resolution by trilinear interpolation:  8  $D_{r}=TI\left(D_{r},4\right)$

The first stage of the algorithmic downsamples the rough disparity map to 1/4 resolution by bilinear interpolation to reduce the computational cost. Then we use the max pool layer to calculate the maximum and minimum values with kernel 3 × 3. After that, we adjust these two values according to the threshold σ and get the average interval for each pixel. Finally, we leverage the average interval, the threshold and minimum value to calculate the disparity range and upsample to the original resolution.

On this basis, we use the bilinear sample (grid_sample) to warp the right features according to the disparity range and concatenate with the left features across each disparity level. Then we can get a simplified 4D cost volume with dimension 1/3H × 1/3W × 8 × 64. Compared to the 4D cost volume with dimension 1/3H × 1/3W × 64 × 64 generated by concatenation directly, the parameters are reduced by eight times, significantly reducing the computational and memory cost.

### 3.5. 3D Stacked Hourglass Module

We construct a 3D stacked hourglass network to optimize the 4D cost volume and regress the disparity, consisting of three hourglass networks. As shown in Figure 1, each hourglass includes two down-sampling and deconvolution upsampling layers. Each down-sampling doubles the number of feature channels. During training, we set different weights for the output of each hourglass to guide the network. The final loss is the weighted sum of the three outputs. During testing, we only use the last hourglass output as the final result. All convolution layers use batch normalization and Relu for non-linearities. A 1 × 1 convolution follows the last layer to decimate the feature dimension. Finally, we use trilinear interpolation to adjust the cost volume to the original resolution and use it for disparity regression.

### 3.6. Disparity Regression

We use the following equation proposed by GCNet [24] for disparity regression. The probability of each disparity *d* is calculated by softmax operation δ(). The final disparity *d* is the weighted sum of each candidate disparity *d* by its probability.

(3) $$\hat{d}=\sum_{d=0}^{D_{max}}d\times \delta \left(-c_{d}\right)$$

For the 2D Disparity Generation Module, the $D_{max}$ is set to 192, while for 3D Stacked Hourglass Module, $D_{max}$ is 24.

### 3.7. Loss Function

We adopt the smooth *L*1 loss function to calculate the loss between all estimated disparities *d* and ground-truth disparities $\hat{d}$, the *L*1 loss is defined as:

(4) $$L\left(d,\hat{d}\right)=\frac{1}{N}\sum_{i=1}^{N}{\mathrm{smooth}}_{L_{1}}\left(d_{i}-{\hat{d}}_{i}\right)$$

in which

(5) $${\mathrm{smooth}}_{L_{1}}\left(x\right)=\left\{\begin{array}{cc} 0.5\times x^{2},\;\mathrm{if}\;\left|x\right|\leq 1 & \\ \left|x\right|-0.5,\;\mathrm{otherwise} \end{array}\right.$$

*L*1 loss function is robust and not sensitive to outliers. The final loss function is the weighted sum of losses over all disparity predictions:

(6) $$L=\sum_{i=1}^{N}\lambda_{i}\times L_{i}$$

We first apply this loss function to 2D and 3D networks respectively to avoid local optimal solution ($\lambda_{2D}=1,\lambda_{3D}=2$). In the fine-tuning step, our network only considers the last output of the 3D stereo network to improve the test accuracy as much as possible ($\lambda_{2D}=0,\lambda_{3D}=1$).

## 4. Experiments

### 4.1. Network Settings and Details

We perform extensive experiments on three popular public data sets to evaluate our proposed JDCNet: Scene Flow [22], KITTI 2012 [35] and KITTI 2015 [36]. We first conduct pre-training on the Scene Flow, then fine-tuning on KITTI 2012 and KITTI 2015. After that, we submit the test results to the KITTI official website to compare with the recent stereo network. We also conduct extensive ablation studies using KITTI 2015 to evaluate the performance difference between 2D and 3D networks.

We implement our network in Pytorch and use Adam as optimizer ($\beta_{1}=0.9$, $\beta_{2}=0.999$). The initial learning rate is set to 0.001. We use a single NVIDIA V100 (NVIDIA, Santa Clara, CA, USA) for training and testing with a batch size of 4. We also conduct data augmentation by cropping the image to 192 × 384.

### 4.2. Results on Scene Flow

Scene Flow [22] is a large scale synthetic dataset. This dataset provides dense disparity maps as ground truth, consisting of 35,454 training and 4370 testing images. The image resolution is 540 × 960. We begin by training for 16 epochs on Scene Flow. The learning rate is $10^{-3}$ for 10 epochs and decays by half after 10, 12 and 14 epoch. We use the best training model to evaluate and compare with other representative stereo networks. We choose EPE ( the end-point error) and running time as the evaluation metrics, where EPE is the mean disparity error of all pixels and running time is the time to process a single stereo pair. The comparison results are shown in Table 2.

As shown in the table, our method has significant improvement in performance with a relatively fast speed. Especially compared with other fast stereo networks, such as AANet [18], DispNetC [22], etc. our method can achieve the best result. Meanwhile, compared with DispNetC and PSMNet [25] that only use the correlation layer or concatenation for matching cost computation and 2D or 3D network for cost aggregation, our JDCNet is significantly better than them. Since we only pre-train on Scene Flow, the performance can be further improved.

### 4.3. Results on KITTI

KITTI is a real-world dataset with street scenes from lidar and a driving car. This dataset provides sparse but accurate disparity maps as ground truth. The image resolution is 376 × 1224. There are 200 stereo image pairs with ground-truth disparities for training and 200 image pairs without ground-truth for testing in KITTI 2012. For KITTI 2015, there are 194 stereo images with ground-truth for training and 195 without ground-truth for testing. We fine-tune on KITTI with the best model on Scene Flow. We combine KITTI 2012 and KITTI 2015 and divide the whole dataset into 370 image pairs for training and 24 image pairs for testing. Both KITTI 2012 and KITTI 2015 have 12 testing images to avoid overfitting. We train 1000 epochs with the learning rate of $10^{-3}$. After that, we further fine-tune on the combined dataset of KITTI 2012 and KITTI 2015 for 300 epochs with the learning rate of $10^{-4}$. Meanwhile, we train 10 epochs with the original high-resolution images and then choose the best model for testing. We evaluate our method using official metrics. We use Out-Noc and Out-All as metrics for KITTI 2012 and D1-bg, D1-fg, D1-all as metrics for KITTI 2015, where Out-Noc and Out-All denote the percentage of erroneous pixels in non-occluded areas and total areas, D1-bg, D1-fg, D1-all denote the percentage of stereo disparity outliers with disparity errors greater than 3 pixels for the background, foreground, and all pixels. Out-All and D1-all are the keys of all metrics. We select some representative 2D and 3D networks for comparison. Table 3 and Table 4 report the results along with running time from the official website.

**Figure 4:**
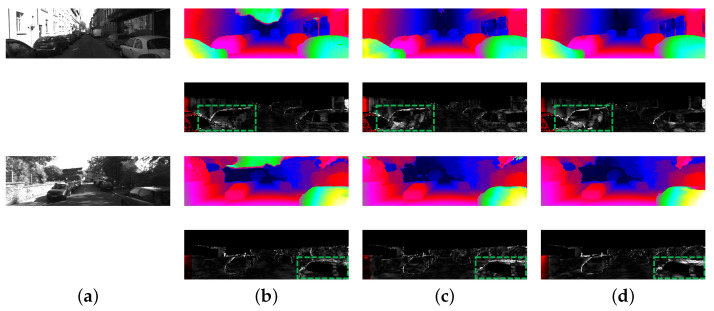
Results of disparity estimation for KITTI 2012 test images. We select the classic 3D stereo method PSMNet and recent proposed fast method AANet for comparison. We use the standard color scheme to represent the error and disparity maps. (**a**) Left Image. (**b**) JDCNet. (**c**) PSMNet. (**d**) AANet.

**Figure 5:**
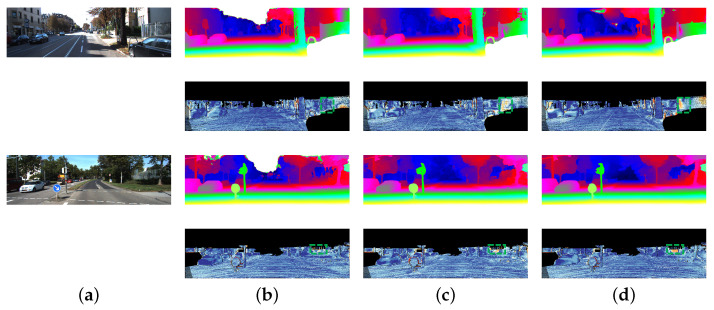
Results of disparity estimation for KITTI 2015 test images. Our method has great advantages in processing foreground pixels, as shown in the green areas. (**a**) Left Image. (**b**) JDCNet. (**c**) PSMNet. (**d**) AANet.

As we can see, compared with other 2D stereo networks (AANet [18], FADNet [20], DispNetC [22], etc.), our method achieves a large improvement on performance, while the running time only increases a little. Compared with other 3D stereo networks, we can get competitive results with a significantly fast speed. In particular, compared with AANet, FADNet and DispNetC, which both use the correlation layer as the same as ours, our method is more accurate than them. Meanwhile, our network achieves similar performance to PSMNet with five times faster. It is because our disparity candidate range is reduced by eight times. By taking advantage of 2D and 3D networks, our network can balance speed and accuracy. Figure 1 can be regarded as a simplified performance comparison with the main metrics and running time for KITTI 2012 and KITTI 2015. Figure 4 and Figure 5 further visualize the disparity result on KITTI 2012 and KITTI 2015 official website.

At the same time, our method can be regarded as a multi-objective optimization programming. The objective functions $f_{1}\left(x\right)$ and $f_{2}\left(x\right)$ are speed and error, respectively. The performance increases as these two metrics decrease. The multiobjective optimization model is defined below:

(7) $$\left\{\begin{array}{cc} V-\min\;F\left(x\right)={\left[f_{1}\left(x\right),f_{2}\left(x\right)\right]}^{T} & \\ x\in X & \\ X\subseteq \mathrm{KITTI} & \end{array}\right.$$

We use pareto dominate to evaluate two decision-making vectors (stereo methods) *a* and *b*. *a* pareto dominate *b*, marked as a>b, if and only if:

(8) $$\left\{\begin{array}{c} \forall i\in \left\{\begin{array}{c} 1,2 \end{array}\right\}f_{i}\left(a\right)\leq f_{i}\left(b\right) \end{array}\right\}\wedge \left\{\begin{array}{c} \exists j\in \left\{\begin{array}{c} 1,2 \end{array}\right\}f_{i}\left(a\right)<f_{i}\left(b\right) \end{array}\right\}$$

Let *S* be the feasible region of multi-objective optimization and f(x) be the vector objective function. If $f\left(X\right)\leq f\left(X^{-}\right)\;\forall X\in S$, then $X^{-}$ is the efficient solution of the multi-objective optimization programming, also called non-dominated solution and pareto optimal solution.

**Figure 6:**
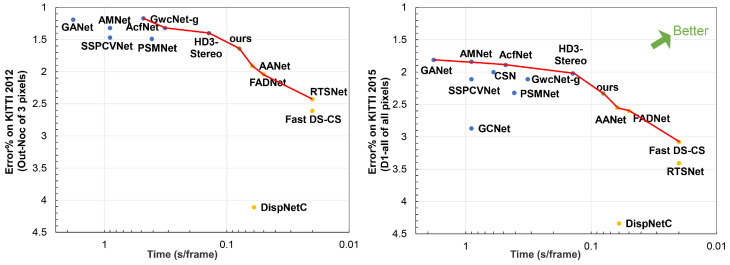
The visualization of pareto front for recent stereo methods. Blue dots represent accurate 3D networks, while yellow dots represent fast 2D networks. The green dot is our proposed method. Our method are the non-dominated solutions for both KITTI 2012 and KITTI 2015. Meanwhile, compared with other fast non-dominated solutions (AANet, FADNet, etc.), our method has the highest accuracy, demonstrating the effectiveness of our proposed method.

For multi-objective optimization problems, there is generally more than one non-dominated solution. All non-dominated solutions form a non-inferior set, also called pareto front, as shown in Figure 6.

As shown in Figure 6, for KITTI 2012, there are seven non-dominated solutions, while our method has the best performance under 100 ms. The same as KITTI 2015, there are eight non-dominated solutions for stere matching, our method can balance 2D networks and 3D networks. All the final results strongly support our idea, a fast and accurate stereo network by combining the 2D and 3D networks.

### 4.4. Ablation Study

We conduct extensive ablation studies on KITTI 2015 with several settings to evaluate our method. We test various combinations of 2D and 3D networks to evaluate the difference between performance and time. First, we use a single 2D network and a 3D network separately for evaluation. We use these two experiments to show the huge difference in speed and performance between 2D and 3D networks. After that, we combine two 2D networks by Algorithm 1, also for 3D networks. We use these two experiments to demonstrate the effectiveness of Algorithm 1. Finally, we combine the 2D network and 3D network by Algorithm 1, the same with our JDCNet. By comparing the performance and running time with other experiments, we can strongly demonstrate the advantages and rationality of our method. The final results are shown in Table 5.

As we can see, the accuracy is far worse than the proposed complementary network if we only use a 2D network. However, if we only use 3D networks, the network needs more parameters and runtime, which is an obstacle to be deployed in real-time applications. This is the same as other 2D and 3D networks. This is because 2D networks lose much feature information when generating 3D cost volumes, while 3D networks need expensive 3D convolutions. Meanwhile, our complementary network achieves the same performance as the 3D network by comparing 2 with 5. However, our method is significantly faster than the 3D network, about five times. Although two cascaded 3D network architecture can achieve a better result than ours, it needs 0.6 s to process a stereo pair, which is similar to other existing 3D stereo methods in terms of accuracy and running time. It is to our advantage that we can trade-off speed and accuracy, with accuracy better than other 2D networks and running time less than other 3D networks, as shown in Figure 1.

## 5. Conclusions

This paper constructs a fast and accurate stereo network that can generate a dense disparity map in 80 ms. We leverage the fast speed of the 2D network and the high accuracy of the 3D network to design a complementary network in a coarse-to-fine manner. We use a 2D encoder-decoder to reduce the disparity candidate range of the 3D network, which can reduce the computational and memory cost simultaneously. Meanwhile, we use a 3D hourglass to improve accuracy. We test our method on three popular benchmarks. The official results demonstrate the effectiveness of our method. Our network can improve performance compared to other 2D networks and is significantly faster than other 3D networks. However, our network’s core idea is to reduce the parameters of the selected network through a 2D network. Thus the biggest disadvantage of our network is that the performance depends on the selected 3D network. So in future work, we attempt to select the best-performing 3D network as the basic network to improve the accuracy as much as possible. Meanwhile, we attempt to use neural architecture search (NAS) to improve the accuracy further.

## Figures and Tables

**Table 1:** Network parameter configuration. H and W are the height and width of the stereo images. The stride of −2 means a deconvolution layer with a stride of 2. Str, Ch (I/O) and CDR are short for Stride, Channel (Input/Output) and classify and disparity regression respectively. Each layer is followed by batch normalization and Relu for non-linearities.

Cascaded Feature Extraction (10 ms)				Hourglass			
Kernel	Str	Ch (I/O)	OutRes	Kernel	Str	Ch (I/O)	OutRes
3 × 3	1	3/32	H × W	3 × 3 × 3	2 1	32/64 64/64	1/6H × 1/6W × 16
5 × 5	3	32/32	1/3H × 1/3W	3 × 3 × 3	2 1	64/128 128/128	1/12H × 1/12W × 32
3 × 3	1	32/32	1/3H × 1/3W	3 × 3 × 3	−2	128/64	1/6H × 1/6W × 16
3 × 3	2	32/48 48/64 64/96 96/128	1/6H × 1/6W 1/12H × 1/12W 1/24H × 1/24W 1/48H × 1/48W	3 × 3 × 3	1	64/64	1/6H × 1/6W × 16
3 × 3	−2	128/96 96/64 64/48 48/32	1/24H × 1/24W 1/12H × 1/12W 1/6H × 1/6W 1/3H × 1/3W	3 × 3 × 3	−2	64/32	1/3H × 1/3W × 8
3 × 3	2	32/48 48/64 64/96 96/128	1/6H × 1/6W 1/12H × 1/12W 1/24H × 1/24W 1/48H × 1/48W	3 × 3 × 3	1	32/32	1/3H × 1/3W × 8
3 × 3	−2	128/96 96/64 64/48 48/32	1/24H × 1/24W 1/12H × 1/12W 1/6H × 1/6W 1/3H × 1/3W	Classify and Disparity Regression			
**2D Disparity Generation (15 ms)**				Kernel	Str	Ch (I/O)	OutRes
Kernel	Str	Ch (I/O)	OutRes	3 × 3 × 3	1	32/32	1/3H × 1/3W × 8
Correlation	-	32/64	1/3H × 1/3W	3 × 3 × 3	1	32/1	1/3H × 1/3W × 8
3 × 3	2	(64/64) × 4	1/6H × 1/6W 1/12H × 1/12W 1/24H × 1/24W 1/48H × 1/48W	Trilinear interpolation	3	8/24	H × W
3 × 3	−2	(64/64) × 4	1/24H × 1/24W 1/12H × 1/12W 1/6H × 1/6W 1/3H × 1/3W	Disparity Regression	1	24/1	H × W
Trilinear interpolation	3	64/192	H × W	**3D Stacked Hourglass (5 ms)**			
Disparity Regression	1	192/1	H × W	Kernel	Str	Ch (I/O)	OutRes
**4D Cost Volume Generation (0.7 ms)**				3 × 3 × 3	1	32/32 32/32	1/3H × 1/3W × 8
Kernel	Str	Ch (I/O)	OutRes	Hourglass	-	-	1/3H × 1/3W × 8
Algorithm 1	-	1/24	H × W	Hourglass	-	-	1/3H × 1/3W × 8
Triliner interpolation	1/3	24/8	1/3H × 1/3W	Hourglass	-	-	1/3H × 1/3W × 8
Warp	-	32/32	1/3H × 1/3W × 8	CDR	-	-	H × W
Concat	-	32/64	1/3H × 1/3W × 8	CDR	-	-	H × W
3 × 3 × 3	1	64/32 32/32	1/3H × 1/3W × 8	CDR	-	-	H × W

**Table 2:** Performance comparison on Scene Flow test set. GCNet, PSMNet, GANet are all 3D stereo networks, while DispNetC and AANet are fast stereo networks. AANet is recent proposed fast and accurate stereo method. Our method is more accurate than other fast (2D) and accurate (3D) stereo methods, also with fast speed.

Method	GCNet [24]	PSMNet [25]	GANet [27]	DispNetC [22]	AANet [18]	JDCNet
EPE	2.51	1.09	0.84	1.68	0.87	0.83
Time(s)	0.9	0.41	1.5	0.06	0.068	0.08

**Table 3:** The official results on KITTI 2012 benchmark. The first four are classic 3D stereo methods, while the others are fast stereo networks. We collect the data from the KITTI offical website. Only published methods are listed for comparison. Bold denotes important metrics or the best results in all fast stereo networks.

Method	>2 pixel		>3 pixel		>5 pixel		Runtime(s)
	Out-Noc	Out-All	Out-Noc	Out-All	Out-Noc	Out-All	
GANet [27]	1.89	2.50	1.19	1.60	0.76	1.02	1.8
GwcNet [31]	2.16	2.71	1.32	1.70	0.80	1.03	0.32
PSMNet [25]	2.44	3.10	1.49	1.89	0.90	1.15	0.41
GCNet [24]	2.71	3.46	1.77	2.30	1.12	1.46	0.9
DispNetC [22]	7.38	8.11	4.11	4.65	2.05	2.39	0.06
RTSNet [19]	3.98	4.61	2.43	2.90	1.42	1.72	0.023
Fast DS-CS [21]	4.54	5.34	2.61	3.20	1.46	1.85	0.021
FADNet [20]	3.27	3.84	2.04	2.46	1.19	1.45	0.05
AANet [18]	2.30	2.96	1.91	2.42	1.20	1.53	0.068
**JDCNet**	2.49	3.13	**1.64**	**2.11**	**1.07**	**1.38**	0.08

**Table 4:** The official results on KITTI 2015 benchmark. The first five are classic 3D stereo methods, while the others are fast stereo networks. Bold denotes important metrics or the best results in all fast stereo networks. Compared with other fast methods, our JDCNet can achieve the best performance in D1-bg and the main evaluation metric D1-all for all and non-occluded pixels.

Method	All Pixels			Non-Occluded Pixels			Runtime(s)
	D1-bg	D1-fg	D1-all	D1-bg	D1-fg	D1-all	
GANet [27]	1.48	3.16	1.81	1.34	3.11	1.63	1.8
CSN [26]	1.86	4.62	2.32	1.71	4.31	2.14	0.41
GwcNet [31]	1.74	3.93	2.11	1.61	3.49	1.92	0.32
PSMNet [25]	1.86	4.62	2.32	1.71	4.31	2.14	0.41
GCNet [24]	2.21	6.16	2.87	2.02	5.58	2.61	0.9
DispNetC [22]	4.32	4.41	4.34	4.11	3.72	4.05	0.06
RTSNet [19]	2.86	6.19	3.14	2.67	5.83	3.19	0.023
Fast DS-CS [21]	2.83	4.31	3.08	2.53	3.74	2.73	0.021
FADNet [20]	2.50	3.10	2.60	2.35	2.61	2.39	0.05
DeepPruner [23]	2.32	3.91	2.59	2.13	3.43	2.35	0.06
AANet [18]	1.99	5.39	2.55	1.80	4.93	2.32	0.068
**JDCNet**	**1.91**	4.47	**2.33**	**1.73**	3.86	**2.08**	0.08

**Table 5:** Evaluation of JDCNet with different settings. We use 182 stereo images of KITTI 2015 for training and remaining 12 images for testing. We select PC3, EPE and running time as the evaluation metrics, where PC3 denote the percentage of three pixel error of all valid pixels, EPE and running time have been introducted in Section 4.2.

	Matching Cost Computation	Cost Aggregation	KITTI 2015		
			PC3 (%)	EPE	Time (s)
1	Correlation	Encoder-Decoder/2D	96.98	0.62	0.02
2	Concat	Hourglass/3D	97.58	0.57	0.41
3	Correlation + Correlation	Encoder-Decoder/2D	97.28	0.59	0.042
4	Concat + Concat	Hourglass/3D	97.78	0.55	0.6
5	Correlation + Concat	Encoder-Decoder/2D + Hourglass/3D	97.56	0.57	0.079

## Data Availability

Not applicable.
